# Endurance Exercise-Induced Fgf21 Promotes Skeletal Muscle Fiber Conversion through TGF-β1 and p38 MAPK Signaling Pathway

**DOI:** 10.3390/ijms241411401

**Published:** 2023-07-13

**Authors:** Xiaomao Luo, Huiling Zhang, Xiaorui Cao, Ding Yang, Yi Yan, Jiayin Lu, Xiaonan Wang, Haidong Wang

**Affiliations:** 1 College of Veterinary Medicine, Shanxi Agricultural University, Jinzhong 030801, China; xmluo@sxau.edu.cn (X.L.); zhlsxau@163.com (H.Z.); 19834542728@163.com (X.C.); 17835424555@126.com (Y.Y.); lionelphilip@126.com (J.L.); 2 College of Veterinary Medicine, China Agricultural University, Beijing 100193, China; yd2020@cau.edu.cn; 3 Renal Division, Department of Medicine, Emory University, Atlanta, GA 30322, USA; xwang03@emory.edu

**Keywords:** endurance exercise, Fgf21, fiber type, TGF-β1, p38 MAPK

## Abstract

Fgf21 has been identified as playing a regulatory role in muscle growth and function. Although the mechanisms through which endurance training regulates skeletal muscle have been widely studied, the contribution of Fgf21 remains poorly understood. Here, muscle size and function were measured, and markers of fiber type were evaluated using immunohistochemistry, immunoblots, or qPCR in endurance-exercise-trained wild-type and *Fgf21* KO mice. We also investigated Fgf21-induced fiber conversion in C2C12 cells, which were incubated with lentivirus and/or pathway inhibitors. We found that endurance exercise training enhanced the Fgf21 levels of liver and GAS muscle and exercise capacity and decreased the distribution of skeletal muscle fiber size, and fast-twitch fibers were observed converting to slow-twitch fibers in the GAS muscle of mice. Fgf21 promoted the markers of fiber-type transition and eMyHC-positive myotubes by inhibiting the TGF-β1 signaling axis and activating the p38 MAPK signaling pathway without apparent crosstalk. Our findings suggest that the transformation and function of skeletal muscle fiber types in response to endurance training could be mediated by Fgf21 and its downstream signaling pathways. Our results illuminate the mechanisms of Fgf21 in endurance-exercise-induced fiber-type conversion and suggest a potential use of Fgf21 in improving muscle health and combating fatigue.

## 1. Introduction

It is widely acknowledged that physical exercise contributes to reducing the risk of numerous physiological and pathological issues, including aging, viral infections, and obesity [1,2]. As a highly dynamic tissue accounting for about 55% of body mass in mammals, the skeletal muscle continuously remodels itself in response to changing functional demands [3]. Unquestionable evidence indicates that resistance and endurance training primarily targeting skeletal muscle enhances its functional capacity in strength, endurance, and metabolism [4,5]. Exercise training of varying intensities can modify the quantity and quality of skeletal muscle’s structural and contractile proteins, based on muscle fiber types and their phenotype [6]. Of these, myosin heavy chain (MyHC) isoforms seem to embody the majority of the fibers’ structural and functional properties. Mammalian skeletal muscles contain four isoforms of MyHC called MyHC IIx, MyHC IIa, MyHC IIb, and MyHC I, which are considered to be type II fast-twitch or glycolytic fibers and type I slow-twitch or oxidative fibers, for instance, MYH1, MYH2, MYH 4, and MYH7 [7,8]. Training-induced skeletal muscle adaptation can cause the shift in fiber types, typically from fast to slow, across a wide range of levels [9]. Recognizing that various types of exercise can induce shifts in muscle fiber type can inform athletic training regimens and aid in rehabilitation settings, particularly when muscle atrophy (muscle wasting) is present. Therefore, identifying different intensities of exercise training and understanding the underlying mechanisms that induce muscle fiber type conversion in mice are crucial.

The fibroblast growth factor (Fgf) family comprises multifunctional signaling molecules with wide-ranging metabolic effects. Fgf21 binds to and activates members of the FgfR superfamily of receptor tyrosine kinases in the target tissues [10]. Previous studies have confirmed that Fgf21 exerts paracrine and endocrine control over numerous aspects of energy homeostasis in multiple tissues [11]. Recently, Fgf21 biology has rapidly expanded to include protective roles against pathologic cardiac hypertrophy, oxidative stress, myocardial infarction, and skeletal muscle atrophy in rodents [12]. Transforming growth factor β (TGF-β) is an evolutionarily conserved family of secreted pleiotropic factors that play critical roles in embryogenesis and adult tissue homeostasis by regulating cell proliferation, differentiation, and cell-specific motility [13,14]. This family includes three isoforms: TGF-β1, TGF-β2, and TGF-β3, all of which exert biological effects by binding to their receptors [15]. Previous studies have shown that TGF-β1 influences the myoblasts’ proliferation and differentiation, fibrosis and inflammation, and the force-generating capacity of skeletal muscle by regulating myogenic regulatory factors during muscle regeneration [16,17,18]. The major mitogen-activated protein kinases (MAPKs), ERK, JNK, and p38 alter cellular physiology in response to extracellular signals, including cell division, inflammation, and differentiation [19]. Indeed, several studies have demonstrated that the p38 MAPK pathway is implicated in myoblast proliferation-to-differentiation at each myogenic stage [20]. Furthermore, it has been reported that the TGF-β/Smad and p38 MAPK signaling pathways participate in regulating skeletal muscle function [21,22]. The presence of Fgfs in different skeletal muscle fiber types and different sexes provides insight into the interaction between the skeletal muscle and other organs [23]. Understanding how muscle fiber types respond to Fgf21 could aid in the development of diets that optimize muscle function and overall health. Simultaneously, increased Fgf21 has been found to inactivate the TGF-β1-Smad2/3-MMP2/9 signaling pathway and alleviate cardiac fibrosis to improve cardiac function in mice with myocardial infarction [24]. However, whether the endurance training adaptation mediated by hepatogenic Fgf21 can shift muscle-fiber-type conversion by regulating TGF-β1/Smad2/3 and p38 MAPK signaling pathway in mice is unclear and warrants further investigation.

In this study, we investigated the roles of Fgf21 on the regulation of endurance-exercise-induced transition of fiber type in gastrocnemius (GAS) muscle, along with the underlying mechanisms in the C2C12 myotubes. We analyzed both the function of skeletal muscle and Fgf21 expression during endurance exercise training, focusing particularly on the GAS muscle fiber type. Utilizing rhFgf21, *Fgf21* KO mice, LV-Fgf21, and signaling pathway inhibitors, we conducted experiments both in vitro and in vivo to explore Fgf21’s role and potential mechanisms in promoting a transition towards slow-twitch muscle fibers in mice undergoing endurance exercise training. Our study provides new insights into the potential application of rhFgf21 in enhancing physical fitness and counteracting fatigue.

## 2. Results

### 2.1. Endurance Exercise Enhances Fgf21 Expression and Exercise Capacity and Shifts Skeletal Muscle Fiber Size Distribution

**Figure 1:**
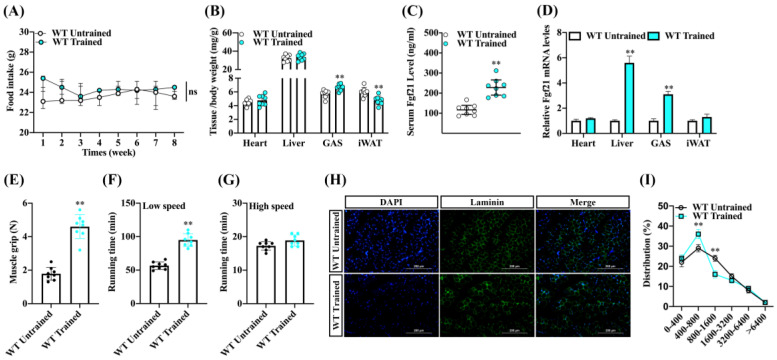
Endurance exercise increased the Fgf21 expression and improves the endurance exercise capacity and fiber conversion of skeletal muscle in mice. Male wildtype C57BL/6 mice were fed a chow diet following endurance exercise training and harvested 8 weeks later. (**A**) Food intake and (**B**) the ratio of heart, liver, GAS, and inguinal white fat to body weight were evaluated. (**C**) Changes in serum Fgf21 concentration and (**D**) the mRNA of Fgf21 in the different tissues were detected. (**E**) The skeletal muscle grip strength, (**F**) running time at low speed, and (**G**) running time at high speed were measured. (**H**) GAS muscle was stained with laminin (scale bar 200 μm) and (**I**) the staining was quantified using ImageJ software V1.8.0. (NIH, Bethesda, MD, USA). The results are reported in the line/bar graph as mean ± SE; *n* = 8/group; Bars mean *p* ≤ 0.05 analyses followed by non-paired Student’s *t*-test. The ns means non-significant (*p* > 0.05); ** *p* < 0.01 vs. WT-untrained.

To ascertain the effects of endurance exercise on skeletal muscle growth, we subjected male wild-type C57BL/6 mice to an endurance exercise training regimen for 8 weeks while feeding them a chow diet (Figure S1). We noted that, aside from an initial increase in food intake at the exercise onset, there were no significant changes to food consumption in the WT-trained mice (Figure 1A). We also observed that WT-trained mice increased the ratio of gastrocnemius muscle (GAS), along with a decreased inguinal white fat to body weight ratio. However, there were no significant differences observed in the heart and liver (Figure 1B). Upon analyzing Fgf21 expression in mice, we found that WT-trained mice had a higher level of Fgf21 in serum than WT-untrained mice. In tandem, WT-trained mice exhibited increased Fgf21 mRNA levels in the GAS and liver (Figure 1C,D). Consistent with the hypothesis that Fgf21 might play a role in stimulating muscle regeneration following muscle injury or damage, we further investigated the roles of endurance exercise training on skeletal muscle contraction properties. We found that endurance exercise training significantly improved muscle grip strength and low-speed running time (Figure 1E,F), but did not influence the high-speed running time (Figure 1G). Interestingly, we also found that endurance exercise training notably increased the proportion of cross-sectional areas (CSA) of the myofibers (400–800 μm^2^), while reducing the proportion of muscle fiber (800–1600 μm^2^) following the training regimen (Figure 1H,I). These results suggest that endurance exercise training could enhance Fgf21 expression and improve exercise performance, potentially through mediated by skeletal muscle fiber conversion, but it appears to not significantly affect explosive performance.

### 2.2. Endurance Exercise Induces Skeletal-Muscle-Fiber-Type Switching

**Figure 2:**
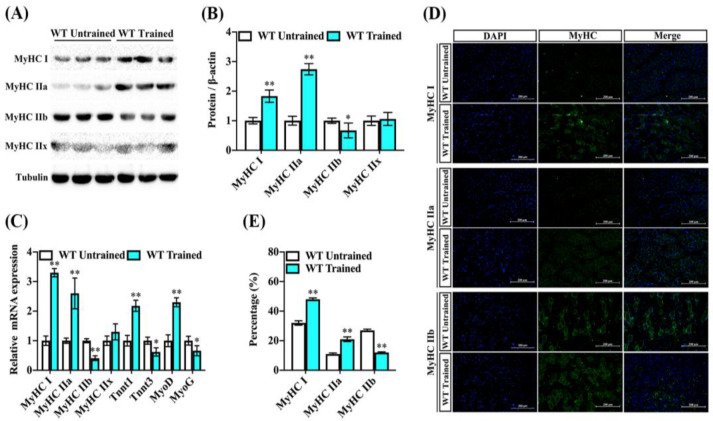
Endurance exercise facilitates fiber-type switching of GAS muscle in mice. Male wildtype C57BL/6 mice were fed a chow diet following endurance exercise training and harvested 8 weeks later. (**A**,**B**) The abundance of MyHC I, MyHC IIa, MyHC IIb, and MyHC IIx proteins in the GAS muscle were measured by immunoblot and analysis for trained and untrained mice. Data are reported in the bar graphs as the fold change of normalized proteins of tubulin (*n* = 6/group; mean ± SE). (**C**) The mRNA expression of MyHC I, MyHC IIa, MyHC IIb, MyHC IIx, TnnT1, TnnT3, MyoD, and MyoG in the GAS muscle via real-time qPCR. Results are reported in the bar graph as the fold changes of individual mRNAs, normalized with GAPDH (*n* = 4/group; mean ± SE). (**D**,**E**) Representative images and quantification of DAPI, MyHC I, MyHC IIa, and MyHC IIb via immunofluorescent staining in the GAS muscle (*n* = 3, Scale bar in 200 μm). Bars mean *p* ≤ 0.05 analyses followed by non-paired Student’s *t*-test. * *p* < 0.05; ** *p* < 0.01 vs. WT-untrained.

Exercise, particularly when considering the type and intensity of training, can indeed induce changes in muscle fiber types. Both myosin heavy chain and troponin isoforms have been associated with skeletal muscle exercise capacity and contraction properties [6]. To evaluate the contribution of endurance exercise training to fiber types in mice, MyHC I, MyHC IIa, MyHC IIb, and MyHC IIx in the gastrocnemius were evaluated via immunoblot analyses and real-time quantitative PCR. The protein levels of muscle-fiber-type MyHC I and MyHC IIa were increased in the GAS muscle of trained mice compared to the untrained mice, whereas the protein level of MyHC IIb was lower in the trained mice’s GAS muscle. Interestingly, the expression of another fiber-type marker remained largely unchanged by endurance exercise training (Figure 2A,B). Similarly, we found that the transcriptions of fiber-associated genes MyHC I, MyHC IIa, and TnnT1 tended to be higher in the GAS muscle of trained mice, while the transcription level of MyHC IIb also showed a reduction. Notably, we found that the expression of MyoD was increased and the MyoG gene was reduced following exercise training, which suggests progression in skeletal muscle formation (Figure 2C). Consistently, the amounts of histologically detectable muscle fiber associated with MyHC I and MyHC IIa were increased in the GAS muscle of trained mice versus untrained mice, but the fluorescence percentage of MyHC IIb was lower compared to untrained mice (Figure 2D,E). These results suggest that endurance exercise training contributes to a transition toward slow-twitch fibers and promotes muscle synthesis in the GAS muscle.

### 2.3. Fgf21 Promotes Fiber-Type Remodeling, Inhibits TGF-β1-Smad2/3-mmp9, and Activates p38 MAPK Signaling Pathway in C2C12 Myotubes

**Figure 3:**
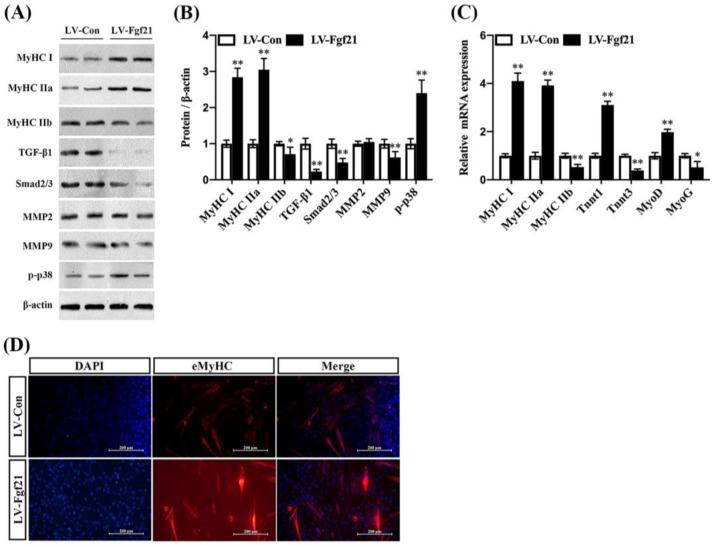
Fgf21 promotes the formation of myotubes and regulates MyHC expression and signaling pathways in C2C12 cells. C2C12 cells were treated with LV-Con and LV-Fgf21 for 12 h and then cultured with 2% horse serum for 5 days. (**A**) Cells were evaluated for MyHC I, MyHC IIa, MyHC IIb, TGF-β1, Smad2/3, mmp2, mmp9, and p-p38 by immunoblot in C2C12 cells. (**B**) Data are shown in the bar graph as the fold change of normalized proteins of β-actin (mean ± SE). (**C**) The mRNA expression of MyHC I, MyHC IIa, MyHC IIb, TnnT1, TnnT3, MyoD, and MyoG for fiber-type and myogenesis markers were detected via real-time qPCR. Results are reported in the bar graph as the fold changes of mRNAs normalized to β-actin (mean ± SE). (**D**) C2C12 cells were evaluated for myotubes eMyHC by immunostaining; DAPI staining was used to determine the number of nuclei. The eMyHC-positive cells are indicated with red color (mean ± SE, Scale bar in 200 μm). Bars mean *p* ≤ 0.05 analyses followed by non-paired Student’s *t*-test. * *p* < 0.05; ** *p* < 0.01 vs. LV-Con.

Fgf21, primarily secreted by the liver and also produced in other tissues including muscle, is known to enhance the function of mitochondria, the cellular components responsible for energy production, potentially impacting muscle fiber type indirectly [25,26]. To investigate the direct role of Fgf21 in regulating muscle fiber types, we performed immunoblot analysis of MyHC I, MyHC IIa, MyHC IIb, TGF-β1, Smad2/3, mmp2, mmp9, and p-p38 proteins in C2C12 cells. We found that neither lentivirus expressing Fgf21 (LV-Fgf21) nor control lentivirus (LV-Con) affected the viability of C2C12 cells after 48 h. However, LV-Fgf21 induced a higher level of Fgf21 than LV-Con in C2C12 cells (Figure S2A–C). Compared to LV-Con, LV-Fgf21 significantly increased the MyHC I and MyHC IIa proteins expression and downregulated the MyHC IIb expression. Importantly, LV-Fgf21 suppressed TGF-β1, Smad2/3, and mmp9 proteins expression, except MMP2. Moreover, the phosphorylation level of p38 was significantly increased in the LV-Fgf21-infected cells (Figure 2A,B). Consistent with these findings, LV-Fgf21 upregulated the slow-twitch fiber marker transcription and suppressed the fast-twitch marker transcription. Additionally, LV-Fgf21 enhanced myogenesis markers in C2C12 myotubes (Figure 2C). Furthermore, we found that LV-Fgf21 could significantly promote the formation of myotubes in C2C12 myotubes (Figure S2D). Like the previous results, we found that LV-Fgf21 accelerated the differentiation of C2C12 cells and led to the formation of more myotubes, as indicated by eMyHC immunofluorescence (Figure 3D). These results suggest that Fgf21 can increase myotubes by improving fiber-type markers, inhibiting TGF-β1-Smad2/3-mmp9, and activating the p38 MAPK signaling pathway in C2C12 myotubes.

### 2.4. Fgf21 Mediates TGF-β1 and p38 MAPK Signaling Pathways Regulating MyHC Expression in C2C12 Myotubes

**Figure 4:**
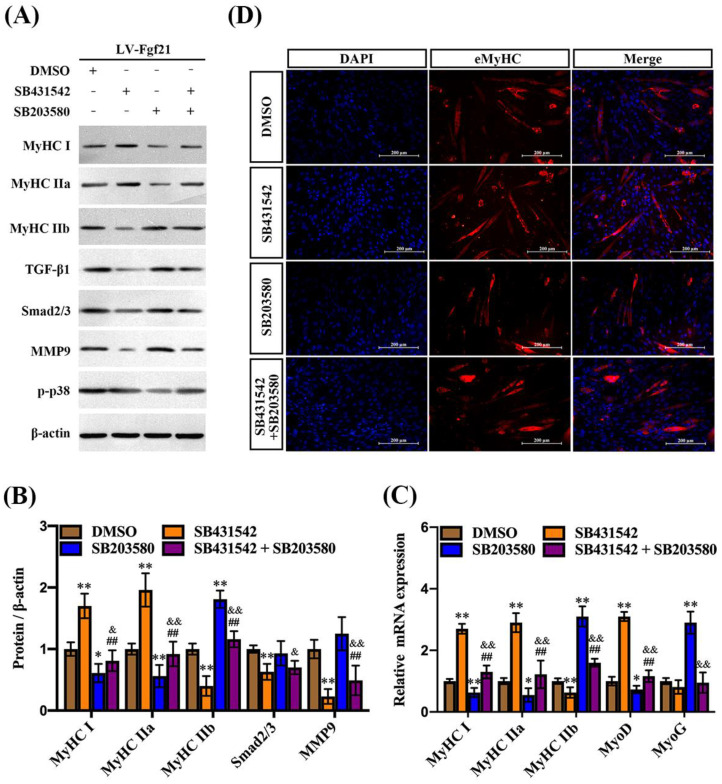
TGF-β1 and p38 MAPK signaling pathways regulate Fgf21-induced MyHC expression in C2C12 myotubes. C2C12 cells were treated with inhibitors for 24 h and then infected with LV-Fgf21 for 12 h, continued induction of myotube differentiation. (**A**) C2C12 cells were harvested to detect MyHC I, MyHC IIa, MyHC IIb, TGF-β1, Smad2/3, mmp9, and p-p38 via immunoblot. (**B**) Data are shown in the bar graph as the fold change of normalized proteins of β-actin (mean ± SE). (**C**) The mRNA expression of MyHC I, MyHC IIa, MyHC IIb, MyoD, and MyoG were detected via real-time qPCR. Results are reported in the bar graph as the fold changes of mRNAs normalized to β-actin (mean ± SE). (**D**) Cellular immunofluorescence staining was used to detect the impact of pathway inhibitors on the eMyHC-positive myotubes induced by Fgf21 (Scale bar in 200 μm). Bars mean *p* ≤ 0.05 analyses followed by one-way ANOVA with post hoc Tukey’s tests. * *p* < 0.05, ** *p* < 0.01 vs. DMSO; ^##^ *p* < 0.01 vs. SB431542; ^&^ *p* < 0.05, ^&&^ *p* < 0.01 vs. SB203580.

TGF-β1 and its downstream Smad2/3 signaling have been implicated in the regulation of muscle mass and function, including the inhibition of muscle growth [27,28]. MMP9, involved in the degradation of extracellular matrix components, plays a role in muscle tissue remodeling, while the activation of p38 MAPK promotes differentiation of muscle cells and formation of myotubes [20,29]. To verify whether Fgf21 regulates MyHC expression and myotube formation by TGF-β1-Smad2/3-MMP9 and the p38 MAPK signaling pathway, C2C12 cells were incubated with signaling pathway inhibitors and then exposed to LV-Fgf21. Following incubation with the TGF-β1 pathway inhibitor, both the protein and mRNA of MyHC I and MyHC IIa were slightly upregulated, while the expression of MyHC IIb was significantly inhibited in the LV-Fgf21-infected cells. Once the TGF-β1 pathway was blocked, the protein and mRNA levels of Smad2/3 and MMP9 were consistently downregulated, yet there was no significant impact on the expression of p-p38. In addition, the p38 pathway inhibitor significantly mitigated the Fgf21-induced upregulation of MyHC I and MyHC IIa levels. The inhibitor only increased the protein level of MyHC IIb, not the mRNA level, in LV-Fgf21-infected cells. When cells overexpressing Fgf21 were incubated with both inhibitors, we found that MyHC IIb was slightly upregulated, while Smad2/3 and MMP9 were downregulated compared to the DMSO incubation (Figure 4A–C). These results suggest that both TGF-β1 and p38 MAPK signaling pathways can be involved in regulating Fgf21-induced changes in MyHC genes, but there is no significant interactive regulation. To further explore the impact of TGF-β1 and p38 MAPK signaling pathways on Fgf21-induced eMyHC, we detected the effects of inhibitors on differentiating cells using immunofluorescence. Compared with DMSO, SB431542 increased the numbers of eMyHC-positive myotubes, while SB203580 exerted an inhibitory effect on positive myotubes. However, when these inhibitors were co-incubated, no significant changes were observed in the number of positive cells (Figure 4D). These results collectively demonstrate that Fgf21 may predominantly regulate MyHC expression via the TGF-β1-Smad2/3-MMP9 and p38 MAPK signaling pathways.

### 2.5. rhFgf21 Counteracts the Inhibitory Effect of Fgf21 KO on Muscle-Fiber-Types of Transition In Vivo

**Figure 5:**
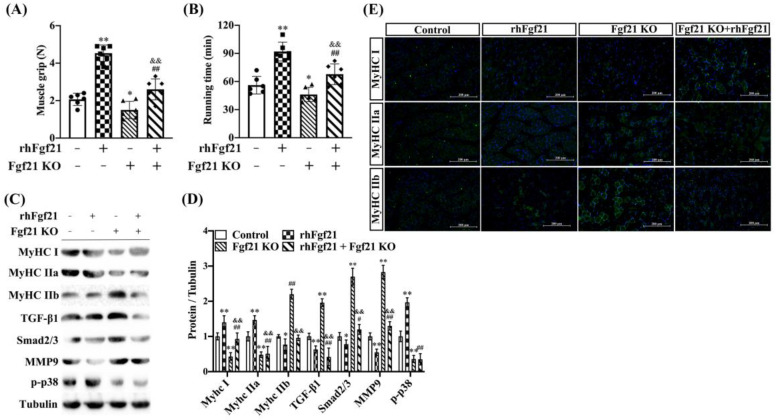
Fgf21 knockout blocks the effect of endurance exercise training on muscle fiber switch. Male wildtype C57BL/6 mice or *Fgf21* KO mice were treated with or without rhFgf21 following endurance exercise training and harvested 8 weeks later. (**A**) The skeletal muscle grip strength and (**B**) running time at low speed were measured (n = 6/group; mean ± SE). (**C**,**D**) The expression of MyHC I, MyHC IIa, MyHC IIb, TGF-β1, Smad2/3, mmp9 and p-p38 proteins were measured in the GAS muscle via immunoblot and analyzed. Data are reported in the bar graphs as the fold change of normalized proteins of Tubulin (n = 4/group; mean ± SE). (**D**,**E**) Representative images of MyHC I, MyHC IIa, and MyHC IIb positive fibers via immunofluorescent staining in the GAS muscle (n = 3, Scale bar in 200 μm). Bars mean *p* ≤ 0.05 analyses followed by one-way ANOVA with post hoc Tukey’s tests. * *p* < 0.05, ** *p* < 0.01 vs. Control; ^#^ *p* < 0.05, ^##^ *p* < 0.01 vs. rhFgf21; ^&&^ *p* < 0.01 vs. *Fgf21* KO.

Consistent with earlier studies, to determine the role of Fgf21 in mediating the transition of GAS muscle fiber types induced by endurance exercise training in vivo, we developed an Fgf21 global knockout mouse model and divided mice into four groups following rhFgf21 as a treatment (Figure S3A,B). Compared to wild-type mice, the muscle grip strength and low-speed running times were reduced in the knockout mice. Notably, treatment with rhFgf21 significantly improved these parameters, effectively counteracting the inhibitory effect of *Fgf21* KO (Figure 5A,B). To evaluate the contribution of rhFgf21 on muscle fiber types and signaling pathways in the *Fgf21* KO mice, we assessed fiber-associated genes and signaling pathways in the GAS muscle through immunoblot analyses and real-time quantitative PCR. MyHC I and MyHC IIa were decreased in the muscles of the *Fgf21* KO mice and when the rhFgf21 was administered, these proteins were increased in muscle to the levels in the control mice. Meanwhile, MyHC IIb, TGF-β1, Smad2/3, and mmp9 were increased in the *Fgf21* KO mice and rhFgf21 treatment almost blocked the upregulation of these proteins, but rhFgf21 had no impact on p-p38 level (Figure 5C,D). Furthermore, the proportions of MyHC I and MyHC IIa muscle fiber types decreased in the GAS muscle of *Fgf21* KO mice compared to controls but increased following treatment with rhFgf21. The proportion of MyHC IIb muscle fibers displayed the opposite trend (Figure 5E). Collectively, these data underscore the indispensable role of Fgf21 in the remodeling of GAS muscle fibers induced by endurance exercise training.

## 3. Discussion

Exercise serves as a potent stimulus for muscle growth and remodeling, enhancing the muscle’s capability to handle various forms of physical stress and improving overall muscular health and function [30]. The intracellular signaling pathways activated by exercise regulate numerous cellular functions, including protein synthesis and degradation, ion balance, energy metabolism, and transcriptional regulation [31]. Cellular adaptations induced by exercise, such as changes in muscle capillary density, mitochondrial homeostasis, satellite cells, and myonuclei, are complex and multi-dimensional [32]. Endurance training, such as long-distance running or cycling, can affect the composition of muscle fiber types. The primary changes typically involve a transition from type IIb fibers (fast, easily fatigued muscle fibers) to type I fibers (slow, fatigue-resistant muscle fibers) or type IIa fibers (intermediate between type I and IIb) [6,33]. Endurance training may induce this transition by enhancing aerobic metabolism, which is vital for sustained endurance performance in the muscles [34]. Interestingly, exercise appears to stimulate the liver to produce Fgf21 and enhance adipose tissue sensitivity to Fgf21 [35,36]. Fgf21 has previously been shown to promote muscle regeneration through the regulation of satellite cells and interact with PGC-1α/AMPK pathway involved in muscle physiology [37,38]. Based on these observations, we speculate that Fgf21 is an indispensable mediator for exercise-induced muscle fiber remodeling. In this study, we found Fgf21 secretion not only increased in the circulatory system, but its expression in skeletal muscle also significantly increased during endurance exercise training. Moreover, endurance exercise training induced the upregulation of myogenic gene expression and slow-twitch muscle fiber gene expression, along with the significant downregulation of fast-twitch muscle fiber gene expression. These observations indicate that Fgf21 may have played a key role in the conversion of GAS muscle fiber types. The ultimate objective of this study is to elucidate the regulatory mechanism of Fgf21 in muscle fiber conversion. By doing so, we aim to establish a scientific foundation for the potential future application of Fgf21 in clinical settings. This could contribute to the protection of muscle health, alleviation of muscle fatigue, and prevention of conditions such as muscle atrophy.

Skeletal muscle fibers are mainly divided into slow-twitch fibers (MyHC I), fast-twitch fibers type IIa (MyHC IIa), mixed fibers type IIb (MyHC IIb), and fast-twitch fibers type IIx (MyHC IIx). Type I and type IIa possess endurance and oxidative metabolic capacity, while MyHC IIb and MyHC IIx exhibit high power and speed, but lower endurance [9]. Fgf21 plays a key role in sugar and fat metabolism, weight regulation, and anti-aging effects [39]. Fgf21 may improve muscle health and function by influencing energy metabolism, repair capacity, and resistance to atrophy in muscles [40]. The focus of Fgf21 has primarily been on its regulatory role in the conversion of skeletal muscle fibers from an energy metabolism perspective [40]. In our study, we discovered that endurance training reduced the cross-sectional area of muscle fibers. Compared to wild-type mice, *Fgf21* KO mice significantly inhibited the changes in the expression of muscle fiber marker genes MyHC I and MyHC IIa at the transcriptional and protein levels, induced by endurance training, while promoting MyHC IIb expression. Simultaneously, our results demonstrate that supplemental injections of rhFgf21 alleviated the inhibitory effect on the expression of slow-twitch fiber marker genes caused by *Fgf21* KO. These findings have contributed to the understanding of the effects of Fgf21 on skeletal muscle fiber types. At the same time, we found that overexpression of Fgf21 significantly promoted the differentiation of myotubes in C2C12 cells and enhanced the expression of eMyHC in myotubes in vitro. MyoD and MyoG, are important indicators of muscle differentiation [41], and Fgf21 showed promoting transcription of MyoD and inhibiting transcription of MyoG, indicating Fgf21 also improves myogenic differentiation. Consistently, we are currently identifying key proteins that interact with Fgf21 to enrich the study of the mechanism of Fgf21’s effect on skeletal muscle fiber types. Taken together, the results have proven that Fgf21’s regulatory effects promote the transformation of fast-twitch fibers into slow-twitch muscle fibers during endurance training.

Troponin T (Tnnt), as an essential muscle protein, is a key component in the mechanism of muscle contraction and part of the actin–myosin complex, which plays a critical role in muscle contraction. In skeletal muscle, Tnnt1 is mainly found in slow muscle fibers, while Tnnt2 and Tnnt3 are found in fast muscle fibers [42]. Therefore, Tnnt expression can significantly impact muscle strength and endurance. In this study, we observed that Tnnt1 was upregulated while Tnnt3 was significantly suppressed. The effects on mRNA of Tnnt1 and Tnn3 were attenuated once rhFgf21 was injected into Fgf21 KO mice. Additionally, Fgf21 overexpression also led to the promotion of Tnnt1 and inhibition of Tnnt3 in the C2C12 myotubes. Interestingly, exercise training improved the grip strength and slow-speed running time of the mice. The *Fgf21* KO mice exhibited significantly inhibiting upregulation of muscle grip strength induced by exercise as well as reduced ability for slow-speed running. Once recombinant rhFgf21 was supplemented, it could reverse the inhibitory effect in the *Fgf21* KO mice. These results demonstrate that the Fgf21-mediated conversion of fast-twitch fibers to slow-twitch fibers by Tnnt1 and Tnnt3 may enhance the muscle’s ability to sustain activity for longer durations without experiencing excessive fatigue.

In the context of fiber type transition, various signaling pathways and factors can influence the expression of myosin heavy chain isoforms, which ultimately defines the fiber type. Skeletal-muscle-fiber-type remodeling involves several crucial signaling pathways, including TGF-β/Smad, MAPK/ERK, and PI3K/AKT [43]. The TGF-β/Smad signaling can influence the differentiation and growth of muscle fibers, thereby playing a role in determining muscle fiber type [44]. However, the precise mechanisms by which TGF-β/Smad signaling regulates muscle fiber type are complex and remain a subject of ongoing research. Previous studies have reported that TGF-β1 can stimulate the phosphorylation of Smad2/3 and affect the differentiation and growth of muscle fibers, suggesting its involvement in muscle fibers regulation [28]. Meanwhile, MMPs participate in muscle repair, and fast- and slow-twitch muscles exhibit different patterns of MMP-9 and MMP-2 activity from Soleus and EDL muscles [45]. In this study, we found that Fgf21 obviously decreased the TGF-β1, Smad2/3, and MMP9 expression in C2C12 myotubes, while there was no noticeable difference in MMP-2 expression. Furthermore, Fgf21 promoted the level of p38 phosphorylation. These data further confirm that Fgf21 can activate TGF-β1-Smad2/3-MMP9 and p38 MAPK signaling pathways. Herein, we further identified TGF-β1-Smad2/3-MMP9 and p38 MAPK signaling pathways to be involved in the regulation of muscle fiber marker genes in LV-Fgf21 infected C2C12 myotubes yet found that the protein and mRNA of MyHC I and MyHC IIa were upregulated, while the expression of MyHC IIb was significantly inhibited during TGF-β1 pathway inhibitor treatment. Meanwhile, the levels of Smad2/3 and MMP9 were significantly downregulated, whereas there was no significant impact on the level of p-p38. Interestingly, the p38 pathway inhibitor showed an obvious inhibitory on the slow-twitch fiber genes but had no effect on the TGF-β1, Smad2/3, and MMP9. These signaling pathway inhibitors also showed the regulation of MyoD and MyoG mRNA activity. Previous research has shown that TGF-β can regulate the expression of specific muscle-fiber-type-related genes through the Smad signaling pathway [28]. During the process of muscle fiber type conversion, MMPs can degrade specific matrix molecules, alter the extracellular environment, and create conditions favorable for the transformation of muscle fiber types [46]. Our findings support the hypothesis that Fgf21 achieves muscle fiber conversion via TGF-β1 through the Smad2/3 pathway, leading to the degradation of MMP9, while also positively activating the p38 signaling pathway. Importantly, Fgf21 does not induce any crosstalk in the regulation of muscle fiber type conversion. In vivo, mice experiment further confirmed the role of the Fgf21-mediated TGF-β1-Smad2/3-MMP9 and p38 MAPK signaling pathway in regulating the transition of fast-twitch fibers to slow-twitch fibers.

## 4. Materials and Methods

### 4.1. Animal Experiments

Wild-type male C57BL/6 mice aged about 12 weeks were purchased from the Experimental Animal Center of Shanxi Provincial People’s Hospital (Taiyuan, Shanxi, China). *Fgf21* KO mice (named C57BL/6N-Fgf21^em1cyage^) were created via CRISPR/Cas-mediated genome engineering (KOCMP-56636-Fgf21-B6N-VA, Cyagen, Suzhou, China). All mice were housed and experimented on in the individual ventilated cage system of the Animal Feeding Center of Shanxi Agricultural University. The room temperature was controlled at 23 °C and mice were given access to food and water freely under 12 h light/dark cycles. All animal care and experimental protocols were performed in accordance with the guide for the Animal Management Rule of the Ministry of Health, People’s Republic of China, and approved by the Animal Medicine Committee of Shanxi Agricultural University (permit numbers: SXAU-EAW-2022M.002107). 

After a week of adaptive training where motorized treadmill (SA101, Nanjing, China) running time (20 min to 60 min) and speed (8 m min^−1^ to 12 m min^−1^) were increased every day, and the inactive mice were culled. C57BL/6 and Fgf21 KO mice with or without 1.5 mg/kg of rhFgf21 (100–42, ThermoFisher, Waltham, MA, USA) for 7 days were randomly divided into 4 groups and were fed with normal standard diets (*n* = 6), respectively. The exercise training mice needed to warm up at the low-speed treadmill at a pace of 8 m min^−1^ for 5 min before the training session. During the endurance exercise training, the speed was set at 8 m min^−1^ for the first two weeks, then increased to 10 m min^−1^ for week 3 and week 4. From the fifth week, the speed was kept at 12 m min^−1^ for the last weeks. The mice were trained for 1 h a day, five days per week for 8 weeks. Importantly, mice were able to complete the endurance exercise training. Body weight and food intake were measured every Sunday at 9 am. After 8 weeks, muscle strength was recorded using a grip test meter system in which a mouse was allowed to hold on to a metal grid with 4 paws and was gently pulled backward by the tail until no longer hold the grid. The running time was detected with the treadmill. Then, mice were sacrificed and serum, heart, liver, gastrocnemius (GAS), and inguinal adipose tissue (iWAT) were weighed, fixed, and frozen.

### 4.2. Wild-Type Generation of Fgf21 Knockout Mouse Model

The *Fgf21* conventional knockout mouse model used in this study was designed and developed with high-throughput electroporation of zygotes by Cyagen Biosciences Inc (Suzhou, China). Briefly, Cas9 mRNA was linearized and purified according to the manufacturer’s instructions. To knockout *Fgf21*, the designed gRNAs’ target sequences were gRNA-A2 (a matching reverse strand of the gene): 5′-GAGTGGGTAACCACGATTGT-TGG-3′ and gRNA-B2 (a matching reverse strand of the gene): 5′-AGAGTCAGGATAAGGTTCCG-GGG-3′. The gRNAs sequences for off-target analysis were 5′-GAGTGGGTAACCACGATTGTTGG-3′ and 5′-AGAGTCAGGATAAGGTTCCGGGG-3′. Furthermore, Cas9 mRNA and gRNAs were co-injected into zygotes of C57BL/6N and transferred to pseudo-pregnant recipients for KO mouse production. The pups genotyped via PCR screening (4375786, ThermoFisher, Waltham, MA, USA) followed by sequencing analysis, and the specific PCR primers (Sangon Biotech, Shanghai, China) are shown in Supplementary Table S1. All the homozygous *Fgf21* knockout mice were produced by male and female F1 heterozygous mice intercrossing and identifying.

### 4.3. ELISA Kit Assays

After mice completed the motorized treadmill training for 8 weeks, blood samples were allowed to clot at room temperature for 30 min prior to serum isolation via centrifugation at 3000 rpm. Serum Fgf21 levels were quantified using a commercial enzyme-linked immunosorbent assay (ELISA) kit (MF2100, R&D, CA, USA) following the manufacturer’s instructions.

### 4.4. Immunofluorescence Staining and Analysis

For staining of muscle sections, mouse GAS muscle was harvested and fixed with 4% paraformaldehyde for 48 h. GAS samples went through dehydration, hyalinization, and paraffin embedding, then 8 μm thick cross-sectional slices were prepared to evaluate the physiological changes of skeletal muscle fibers. The sections were incubated in 0.01 M PBS containing 0.5% Triton-X (PBS-T) and treated with 5% bovine serum albumin (BSA) for 1 h, then incubated with primary antibodies including rabbit anti-Laminin (PA1-16730, Invitrogen, Carlsbad, CA, USA), anti-MyHC I (22280-1-AP, Proteintech, Wuhan, China), rabbit anti-MyHC IIa (ab124937, Abcam, Cambridge, UK), rabbit anti-MyHC IIb (20140-1-AP, Proteintech), and mouse anti-MyHC IIx (67299-1-Ig, Proteintech) at 4 °C overnight. On the next day, the sections were incubated with goat anti-rabbit Alexa Fluor 488 (ab150077, Abcam) and goat anti-mouse Alexa Fluor 488 (ab150113, Abcam) secondary antibodies in darkness at room temperature for 1 h. Then, slides were washed with PBS three times and sealed with anti-fluorescence attenuation sealing tablets containing DAPI (S2110, Solarbio, Beijing, China). Images of fluorescent intensity were captured with a fluorescence microscope (Nikon Eclipse Ts-2R, Tokyo, Japan). Image J software V1.8.0. (National Institutes of Health, Bethesda, USA) was used to analyze the cross-sectional area (CSA) of muscle fibers. CSAs for these samples are reported in μm^2^.

For staining of LV-Fgf21-infected cells or inhibitors-incubated C2C12 cells, C2C12 cells were cultured with slide and fixed with cold 4% paraformaldehyde for 30 min. Then, cultured cells were punched with 0.1% Triton for 10 min and incubated in goat serum blocking solution for 1 h under shaking at room temperature. After washing with PBS, the cells were immunoassayed with mouse anti-eMyHC antibody (SC-53091, Santa Cruz, CA, USA) overnight in the wet box and then incubated with Alexa Fluor 594 (ab150116, Abcam) secondary antibody at room temperature for 1 h. Finally, the slides were treated with DPAI, and a fluorescence microscope was used to take photos.

### 4.5. Western Blotting

Frozen GAS and C2C12 cells were homogenized in RIPA buffer (R0010, Solarbio) containing 1 mM PMSF (P0010, Solarbio), and protease inhibitor cocktail (539133, Merck, Rahway, NJ, USA). The protein concentration was measured using a BCA protein assay kit (P0012, Beyotime, Shanghai, China). Equivalent proteins were subjected to sodium dodecyl sulfate-polyacrylamide gel electrophoresis and transferred to polyvinyl difluoride (PVDF) membranes (ISEQ00010, Merck). After blocking the membrane with 5% non-fat dry milk for 1.5 h, primary antibodies were proceeded overnight at 4 °C and included rabbit anti-Fgf21 (ab171941, Abcam), rabbit anti-MyHC I (22280-1-AP, Proteintech), rabbit anti-MyHC IIa (ab124937, Abcam), rabbit anti-MyHC IIb (20140-1-AP, Proteintech), and mouse anti-MyHC IIx (67299-1-Ig, Proteintech), rabbit anti-TGF-β1 (bs-0086R, Bioss, Beijing, China), rabbit anti-Smad2/3 (PA5-99539, Invitrogen), rabbit anti-p-p38 MAPK (8690, CST, MA, USA), mouse anti-Tubulin (T6199, Sigma-Aldrich, MO, USA), and mouse anti-β-actin (4967, CST). HRP-conjugated goat anti-mouse (CW0102S, CWBIO, Taizhou, China) or anti-rabbit (CW0156S, CWBIO) secondary antibodies were incubated at room temperature for 1 h. ECL (PEOO10, Solarbio) was used for enhanced chemiluminescence detection, according to the manufacturer’s instructions.

### 4.6. RNA Extraction, Reverse Transcript, and Quantitative Real-Time PCR

Total RNA of GAS and treated C2C12 cells were isolated by homogenizing samples in TRIzol™ reagent (15596018, Invitrogen) as described by the manufacturer. The concentration and purity of total RNA were measured using a NanoDrop spectrophotometer (ThermoFisher) and RNA was reverse transcribed into cDNA using M-MLV reverse transcriptase with 2 µg RNA initially treated with DNase I, random primer, first-strand buffer, RNase inhibitor, and DTT (18064071, ThermoFisher). Quantitative PCR was performed with Mix SYBR (Q711-02, Vazyme, Nanjing, China) using Biosystems QuantStudio5 real-time PCR System and quantified using the ∆∆Ct method [47]. Primer sequences are listed in Supplementary Table S2.

### 4.7. Cell culture and Differentiation

The mouse myoblast cell line C2C12 (CRL-1772) was purchased from American Type Tissue Culture (ATCC) and was cultured in high glucose Dulbecco’s modified Eagle’s medium (DMEM) supplemented with 10% heat-inactivated fetal bovine serum (11011, Tianhang, Hangzhou, China), 100 U/mL penicillin, and 100 μg/mL streptomycin in a fully humidified atmosphere containing 5% CO2 at 37 °C. When C2C12 cells were seeded and reached 70% confluency, the medium was replaced with DMEM containing 10 μM SB431542 (S1067, Selleck, Houston, TX, USA) and/or 20 μM SB203580 (S8307, Sigma-Aldrich) for 24 h, and then cultured cells with 2% horse serum DMEM to induce C2C12 cells’ differentiation to myotube.

### 4.8. Lentiviral Particle Preparation and Treatment

The full-length *Fgf21* (Accession no. NM_020013.4) was cloned into PCDH-CMV-MCS-EF1-copGFP vector according to the manufacturer’s instructions and packaged into lentivirus, as in our previous study [48]. PCR primer sequences are listed in Supplementary Table S1. Recombinant plasmid, psPAX2, and PMD2-VSV plasmids were co-transfected to HEK-293T cells for 48 h to harvest supernatants, then the lentiviral particle was concentrated and verified the titer of the virus. C2C12 cells were allowed to reach 50% confluency overnight, and the medium with 100 MOIs of LV-Con- or LV-Fgf21-infected cells for 12 h. After refreshed medium, C2C12 cells induced differentiation into myotubes.

### 4.9. Statistical Analysis

The data were presented as means ± SE. A one-way analysis of variance (ANOVA) was conducted to assess multiple groups, while comparisons between 2 groups were performed by unpaired Student’s test. *p* values for multiple comparisons were followed by Bonferroni post hoc test. Statistical significance was defined as *p* < 0.05.

## 5. Conclusions

In summary, the data in this study provide compelling evidence of Fgf21’s role in the transformation from fast-twitch to slow-twitch fiber types in gastrocnemius muscle, induced by endurance exercise training. Our findings demonstrate that Fgf21 utilizes the TGF-β1-Smad2/3-MMP9 and p38 MAPK signaling pathways in this regulatory process. A deeper understanding of Fgf21’s regulatory mechanisms in C2C12 myotubes could offer new insights into the potential use of rhFgf21 to improve physical fitness and counteract fatigue.

## Data Availability

The data that support the findings of this study are available from the corresponding author upon reasonable request.

## Supplementary Materials

The following supporting information can be downloaded at: https://www.mdpi.com/article/10.3390/ijms241411401/s1.
