# Supplementary material for: Endurance Exercise-Induced Fgf21 Promotes Skeletal Muscle Fiber Conversion through TGF-β1 and p38 MAPK Signaling Pathway
Source: Int J Mol Sci. 2023 Jul 13;24(14):11401. doi: 10.3390/ijms241411401 (PMC10379449; doi:10.3390/ijms241411401)
Supplement: Supplementary file 1 [file ijms-24-11401-s001.zip › ijms-2493120-supplementary.pdf]

Supplementary Material

Exercise-induced Fgf21 promotes skeletal muscle fiber conversion through TGF-β1 and p38 MAPK signaling pathway

Xiaomao Luo[1], Huiling Zhang[1], Xiaorui Cao[1], Ding Yang[2], Yi Yan[1], Jiayin Lu[1], Xiaonan Wang[3], Haidong Wang[1*]

*** Correspondence:**

wanghaidong@sxau.edu.cn

1 Supplementary Figures and Tables

1.1 Supplementary Figures

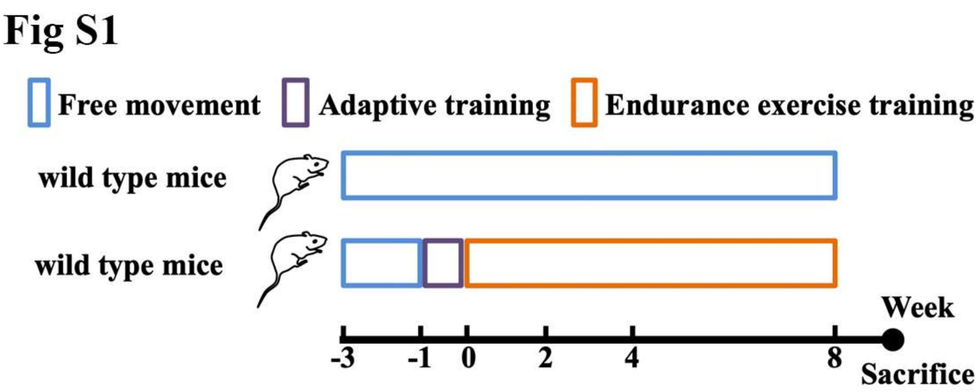

Fig S1. The wild-type mouse endurance exercise training mode diagram. Wild-type mice are divided into 2 groups. After free movement and adaptive training for 4 weeks, mice undergo endurance exercise training lasting 8 weeks.

Fig S2

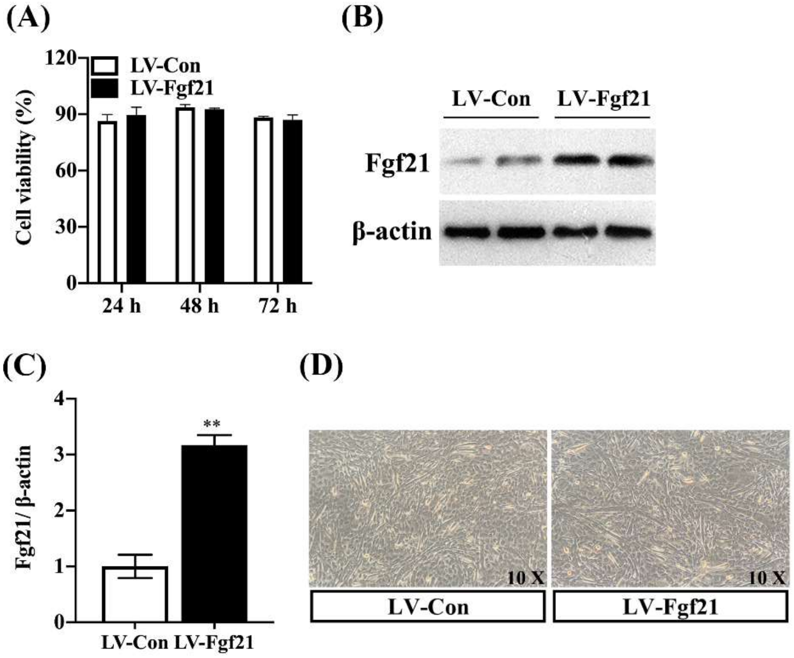

Fig S2. Overexpression of Fgf21 and Fgf21 promotes myogenesis in C2C12 cells. (A) C2C12 cells were respectively infected with 100 MOIs of lentivirus for 24 h, 48 h, and 72 h. The cell viability was detected by CCK-8. **(B)** C2C12 cells were infected with lentivirus and then analyzed the levels of Fgf21 were by western blotting. **(C)** The analysis was reported in the bar graph as the fold change of each protein, normalized to the β-actin. **(D)** C2C12 cells were treated with LV-Con and LV-Fgf21 lasting 12 h, changed the medium with 2% horse serum for 5 days, and light microscopy observed the differentiation of myotubes. Bars mean $P \leq 0.05$ analyses followed by non-paired Student's t-test. $^{**}P < 0.01$ vs. LV-Con.

Fig S3

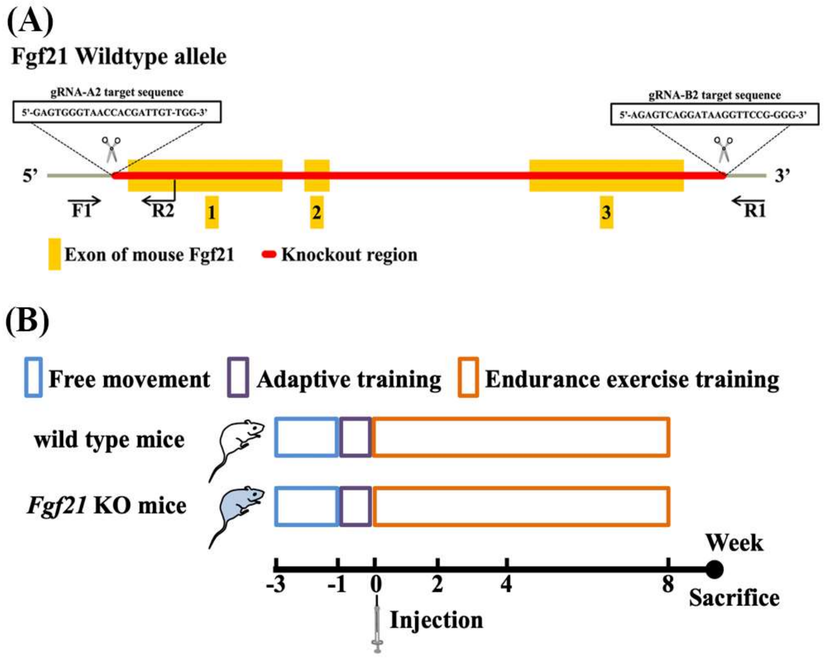

Fig S3. The mode diagrams of *Fgf21* knockout mice genotyping strategy and wild-type or *Fgf21* KO mice experimental design. (A) The *Fgf21* gene (NCBI Sequence: NM_020013) was used to produce KO mice by co-injecting Cas9 and gRNA into fertilized eggs. **(B)** Experiment procedure: Mice were divided into 4 groups: wild-type mice, wild-type mice with rhFgf21 treatment, *Fgf21* KO mice, and *Fgf21* KO mice with an injection of rhFgf21, once per week.

TABLE S1 | Sequences of the primers used in this study.

Gene Primer	Primer sequence (5’-3’)
Genotype-*Fgf21*_F	CAGACCCAGGAGTGTAGACTTCAG
Genotype-*Fgf21*_R	CCAGTGGTTCCATTCTCAGTAC
LV-Fgf21_F	CCGGAAGAGATGGAATGGATGAGATCTAGAGTTGG
LV-Fgf21_R	CCGGAATTCTCAGGACGCATAGGATGCC

TABLE S2 | Primers for Q-PCR in this study.

Gene Primer	Primer sequence (5'-3')
Fgf21_qPCR_F	ACCTGGAGATCAGGGAGGAT
Fgf21_qPCR_R	GTCCTCCAGCAGCAGTTCTC
MyHC I_qPCR_F	AGAGCCAAGAGGAAACTGGAGG
MyHC I_qPCR_R	CTCGTCCTCAATCTTGCTCTGC
MyHC IIa_qPCR_F	ATGAGCTCCGACGCCGAG
MyHC IIa_qPCR_R	TCTGTTAGCATGAACTGGTAGGCG
MyHC IIb_qPCR_F	TGATCACCACCAACCCAT
MyHC IIb_qPCR_R	CAGCCTTGTCAGCAACTTC
MyHC IIx_qPCR_F	AAGGAGCAGGACACCAGCGCCCA
MyHC IIx_qPCR_R	ATCTCTTTGGTCACTTTCCTGCT
Tnnt1_qPCR_F	ACTGCCTCCCGTAAACTCATG
Tnnt1_qPCR_R	TCCTCATCCACCACCTCTACC
Tnnt3_qPCR_F	CCCTCATTGACAGCCACTT
Tnnt3_qPCR_R	CAATCTGTTCTGGCGTTCC
MyoD_qPCR_F	GAATGGCTACGACACCGCCTACTAC
MyoD_qPCR_R	ACGGGGTCTGGGTTCCCTGTT
MyoG_qPCR_F	AACTACCTTCCTGTCCACCTTC
MyoG_qPCR_R	CACAGACTTCCTCTTACACACCT
β-actin_qPCR_F	GCGCGGCTACAGCTTCACCA

β-actin_qPCR_R	GGGCAGCGGAACCGCTCATT
